# Dissection of Antibody Responses of Gam-COVID-Vac-Vaccinated Subjects Suggests Involvement of Epitopes Outside RBD in SARS-CoV-2 Neutralization

**DOI:** 10.3390/ijms24065104

**Published:** 2023-03-07

**Authors:** Maria Byazrova, Pia Gattinger, Ekaterina Astakhova, Gerhard Hofer, Musa Khaitov, Alexander Filatov, Rudolf Valenta

**Affiliations:** 1 National Research Center—Institute of Immunology FMBA of Russia, 115478 Moscow, Russia; 2 Department of Immunology, Faculty of Biology, Lomonosov Moscow State University, 119991 Moscow, Russia; 3 Department of Immunology, Peoples’ Friendship University of Russia (RUDN University) of Ministry of Science and Higher Education of the Russian Federation, 117198 Moscow, Russia; 4 Division of Immunopathology, Department of Pathophysiology and Allergy Research, Center for Pathophysiology, Infectiology and Immunology, Medical University of Vienna, 1090 Vienna, Austria; 5 Department of Materials and Environmental Chemistry, University of Stockholm, 106 91 Stockholm, Sweden; 6 Department of Immunology, Pirogov Russian National Research Medical University, 117997 Moscow, Russia; 7 Department of Clinical Immunology and Allergy, Sechenov First Moscow State Medical University, 119435 Moscow, Russia; 8 Karl Landsteiner University of Health Sciences, 3500 Krems, Austria

**Keywords:** SARS-CoV-2, COVID-19, Gam-COVID-Vac, epitope, antibody, virus-neutralization, molecular interaction assay, Omicron

## Abstract

Millions of people have been vaccinated with Gam-COVID-Vac but fine specificities of induced antibodies have not been fully studied. Plasma from 12 naïve and 10 coronavirus disease 2019 (COVID-19) convalescent subjects was obtained before and after two immunizations with Gam-COVID-Vac. Antibody reactivity in the plasma samples (n = 44) was studied on a panel of micro-arrayed recombinant folded and unfolded severe acute respiratory syndrome coronavirus 2 (SARS-CoV-2) proteins and 46 peptides spanning the spike protein (S) and by immunoglobulin G (IgG) subclass enzyme-linked immunosorbent assay (ELISA). The ability of Gam-COVID-Vac-induced antibodies to inhibit binding of the receptor-binding domain (RBD) to its receptor angiotensin converting enzyme 2 (ACE2) was investigated in a molecular interaction assay (MIA). The virus-neutralizing capacity of antibodies was studied by the pseudo-typed virus neutralization test (pVNT) for Wuhan-Hu-1 and Omicron. We found that Gam-COVID-Vac vaccination induced significant increases of IgG_1_ but not of other IgG subclasses against folded S, spike protein subunit 1 (S1), spike protein subunit 2 (S2), and RBD in a comparable manner in naïve and convalescent subjects. Virus neutralization was highly correlated with vaccination-induced antibodies specific for folded RBD and a novel peptide (i.e., peptide 12). Peptide 12 was located close to RBD in the N-terminal part of S1 and may potentially be involved in the transition of the pre- to post-fusion conformation of the spike protein. In summary, Gam-COVID-Vac vaccination induced S-specific IgG_1_ antibodies in naive and convalescent subjects in a comparable manner. Besides the antibodies specific for RBD, the antibodies induced against a peptide close to the N-terminus of RBD were also associated with virus-neutralization.

## 1. Introduction

After the first cases of infection with the Severe Acute Respiratory Syndrome Coronavirus 2 (SARS-CoV-2) at the end of 2019 in Wuhan, China, the novel corona virus disease (COVID-19) quickly spread all over the world and grew into a global pandemic [1,2,3,4]. The fast development of COVID-19 vaccines and introduction of global vaccination programs together with the appearance of less pathogenic SARS-CoV-2 variants are considered as significant factors for the reduction of COVID-19-associated deaths and the severity of the pandemic [1,5]. The first vaccines which became available were genetic vaccines based on adenovirus-mediated transfer of the gene coding for the SARS-CoV-2 surface protein S [6,7,8]. The SARS-CoV-2 S protein consists of the S1 subunit containing the receptor binding domain (RBD) attaching to its receptor ACE2 on human cells and the S2 subunit that ensures virus attachment to the host cell membrane by proteolytic cleavage of the fusion peptide (FP) during the virus infection [9]. The S gene used for gene transfer in certain adenovirus-based vaccines (e.g., COVID-19 Vaccine Janssen) may be modified to stabilize the protein and to influence its cleavage but this is not the case for the others [10]. Human cells which are infected by adenoviruses containing the S-encoding gene produce to a varying amount the S antigen which is released and presented by the major histocompatibility complex (MHC) class II to T cells resulting in CD4^+^ T cell help for the production of S-specific antibodies. S protein expressed by transfected cells is also presented by MHC class I on the surface of the infected cell and triggers the activation of S-specific cytotoxic CD8^+^ T cells, which can recognize S antigen-producing cells and destroy them. The principle of vaccination, i.e., the introduction of S-encoding genetic information into host cells and subsequent production of S antigen by the infected cells, is similar for messenger ribonucleic acid (mRNA) vaccines, albeit the technology of the gene transfer differs from adenovirus-based vaccines. Thus, “genetic vaccines” mimic to some extent a natural SARS-CoV-2 infection.

The identification of epitopes recognized by antibodies from vaccinated subjects involved in virus neutralization is crucial not only for understanding the mechanism of the action of existing vaccines but also for the development of refined active vaccination and passive immunization strategies against COVID-19 [11,12]. Most of the current vaccines are focused on obtaining an S-specific antibody response with special attention to RBD-specific antibody response, because it has been seen that RBD-specific antibodies are correlated with high neutralizing activity against SARS-CoV-2 [13,14,15]. However, the number of mutations acquired by each new variant of concern (VOC), indicates the importance of identifying epitopes conserved among VOCs targeted by neutralizing antibodies which are located not only in RBD but also outside of RBD [16]. In fact, there are studies indicating the presence of neutralizing epitopes outside RBD, which are recognized by antibodies after immunization [17,18,19,20].

In 2020 the vector-based vaccine Gam-COVID-Vac, also termed Sputnik-V, became available for use in clinical practice and currently has been approved for use in more than 70 countries (https://sputnikvaccine.com/about-vaccine/ (accessed on 17 January 2023)). According to Our World In Data (OWID) resource, 79 million people in Russia received at least one shot of the vaccine and 72 million are fully vaccinated (https://ourworldindata.org/covid-vaccinations?country=RUS (accessed on 22 March 2022)). Gam-COVID-Vac consists of two vaccine types, which are based on two different human adenovirus vectors (hAd26 and hAd5) carrying the native form of the S-protein of the Wuhan-Hu-1 strain SARS-CoV-2. Sputnik-V comprises two injections, one with hAd26 and a second with hAd5 and Sputnik light, with only hAd26. The two different adenovirus vectors are used for the first and second immunization to reduce the possibility that vaccinated subjects produce besides immunity to the S protein also immunity against the adenovirus. In fact, adenovirus-specific antibodies would limit the efficacy of booster vaccinations by neutralization of the adenovirus vector vehicle. Therefore, on the 21st day after the first dose administration of vaccine with rAd26, repeated antigen delivery is carried out with the rAd5 vector [8,21]. Few studies have investigated to any appreciable extent S- and RBD-specific antibody responses and the virus neutralization activity in sera from Gam-COVID-Vac vaccinees [10,22,23]. Increases of RBD-specific IgG antibodies three months after Gam-COVID-Vac administration have been reported and it has been shown that antibodies cross-react with Alpha, Beta, Delta, and Omicron variants of concern [24,25,26]. Gam-COVID-Vac has also been compared with several other licensed vaccines against SARS-CoV-2, which showed high virus-neutralization and high effectiveness against COVID-19-related mortality for Sputnik-V, although slight inferiority to some mRNA vaccines regarding virus-neutralization was found [24]. Finally, it has been shown that immunization of human immunodeficiency virus positive (HIV+) antiretroviral therapy-treated (ART-treated) patients with Gam-COVID-Vac shows epidemiological efficacy against the original wild type and Delta variant of SARS-CoV-2 [27].

However, so far no detailed analysis of the epitope recognition by antibodies in Gam-COVID-Vac vaccinated subjects has been performed and data are needed to understand the immunoglobulin isotype and IgG subclass responses in Gam-COVID-Vac-vaccinated subjects. Here we investigated two groups of subjects who had been vaccinated with Gam-COVID-Vac, one who had had a previous SARS-CoV-2 infection and another group of SARS-CoV-2 naïve subjects. We performed a meticulous analysis of the epitope specificity of induced antibodies using micro-arrayed SARS-CoV-2-derived folded and unfolded proteins and peptides spanning the complete S protein, including RBD. Furthermore, IgG subclass responses were analyzed. Our study reveals that Gam-COVID-Vac-vaccinated subjects mount RBD-specific antibodies capable of blocking the RBD-ACE2 interaction. Interestingly, we found that virus neutralization was also associated with antibodies directed against a peptide outside RBD, which may be involved in the transition of the pre- to post-fusion conformation of the spike protein.

## 2. Results

### 2.1. Gam-COVID-Vac-Vaccinated Subjects Include COVID-19 Naïve and Convalescent Subjects

Convalescent subjects were infected by the Wuhan-Hu-1 strain according to its prevalence and sequencing data obtained at the time of infection in the Moscow region. Naïve subjects had no symptoms of COVID-19 and lacked SARS-CoV-2-specific antibodies at the time of recruitment. Study subjects included 15 females and 7 males with a median age of 60 (i.e., 25–70 years) (Table 1).

**Figure 1:**
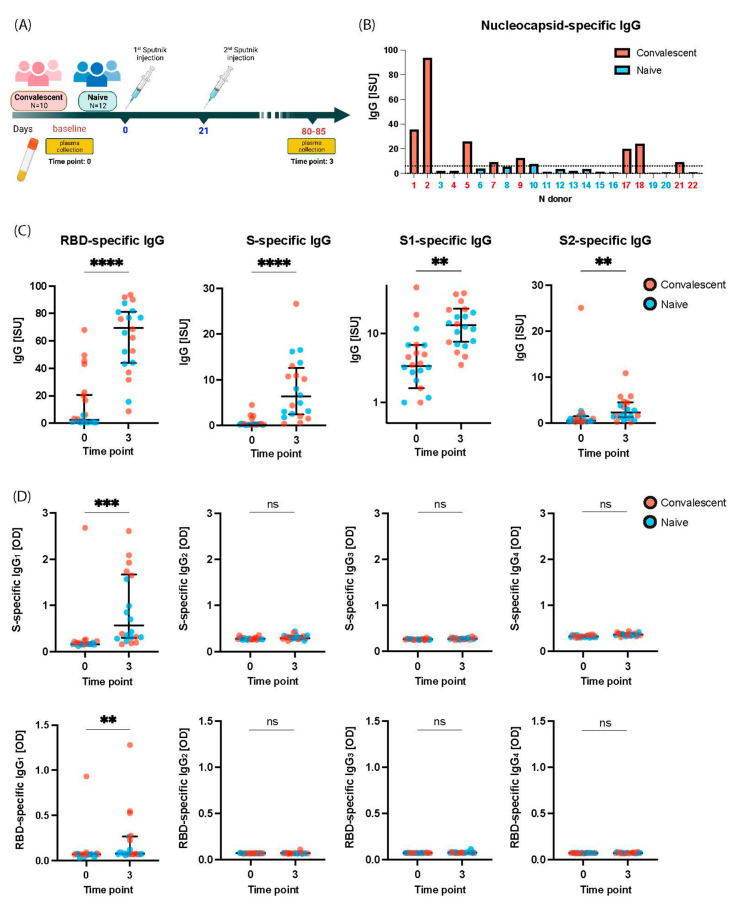
Study design and SARS-CoV-2-specific antibody responses before and after vaccination with Sputnik-V. (**A**) Study design. Plasma samples were obtained from COVID-19-naïve (n = 12) and convalescent subjects (n = 10) at time point 0 (baseline), then subjects received two Sputnik-V vaccinations at time points 1 and 2, respectively and a plasma sample was obtained at time point 3 (i.e., day 80–85 after the first vaccination). (**B**) Nucleocapsid-specific IgG antibody levels (*y*-axis: ISU) in naïve and convalescent subjects (*x*-axis) at baseline. The horizontal line indicates the cut-off for positivity. (**C**) IgG antibody levels (*y*-axes: ISU) specific for RBD, S, S1, and S2 in subjects at time point 0 and 3 (*x*-axes). (**D**) From left to right IgG_1_, IgG_2_, IgG_3_, and IgG_4_ antibody levels (*y*-axes: optical density OD values) to S (upper part) and RBD (lower part) at time points 0 and 3 (*x*-axes). Significant differences between groups as determined by Wilcoxon test are indicated: ** *p* < 0.01, *** *p* < 0.001, **** *p* < 0.0001, ns—not significant.

A plasma sample was obtained from each of the subjects at time point 0 (i.e., baseline) (Figure 1A), then each subject was vaccinated with Sputnik-V with an interval of approximately 21 days between the first and second injection (time points 1 and 2). At time point 3 a second plasma sample was collected to determine vaccine-induced antibody responses (Figure 1A). Subjects were observed for an additional 120 days. On day 110 after final vaccination one subject (i.e., subject 22) had COVID-19 whereas the others had not developed any symptoms of COVID-19 (Table 1).

### 2.2. Gam-COVID-Vac Vaccination Induces Broad IgG Responses to S Including S1, RBD, and S2

We performed a detailed analysis of antibody responses towards a large panel of micro-arrayed folded and unfolded SARS-CoV-2 proteins and peptides spanning the S protein in plasma samples from SARS-CoV-2-naïve and convalescent subjects after a full course of immunizations with Gam-COVID-Vac (Figure 1A,B; Table 1). When comparing plasma samples from time point 0 and time point 3 after vaccination, a significant induction of IgG responses against folded but not unfolded RBD, S, S1, and S2 was found (Figure 1C). Three months after vaccination subjects showed a 21-fold increase of RBD-specific IgG antibodies (*p* < 0.0001) and a 20-fold increase of S-specific IgG antibodies (i.e., time point 0: median 0.291, time point 3: median 5.778) (*p* < 0.0001), respectively. IgG antibodies to S1 increased 3.7-times (*p* = 0.0023) and IgG antibodies to S2 increased 2.8-times (*p* = 0.0094) (Figure 1C).

Vaccination with Gam-COVID-Vac induced mainly IgG responses to folded S, S1, and RBD in a comparable manner in naïve and convalescent subjects (Figure 1C, Figures S2 and S3), indicating that it can be used to boost SARS-CoV-2-specific antibody responses also in convalescent subjects. Significant boosts of IgG antibodies to RBD, S, and S1 were observed in naïve and convalescent subjects (Figure S2) and there were no significant differences regarding IgG levels to RBD, S, S1, and S2 in naïve and convalescent subjects at time point 3 (Figure S3).

### 2.3. Gam-COVID-Vac Vaccination Induces Mainly an IgG_1_ Subclass Response to S and RBD

It has been reported that allergen-specific immunotherapy (AIT) induces mainly allergen-specific IgG_1_ and IgG_4_ responses which differ not only regarding their effector functions (IgG_1_ can mediate antibody-dependent cellular cytotoxicity (ADCC) and complement activation whereas IgG_4_ does not have these effector functions) but also regarding their kinetics (IgG_1_: rapid onset and early decline: IgG_4_: slow onset but sustained levels) [28,29,30]. We therefore investigated the IgG subclass (i.e., IgG_1_–IgG_4_) responses to S and RBD in Sputnik-V-vaccinated subjects. Figure 1D shows that vaccination induced almost exclusively an IgG_1_ subclass response to S and RBD. Significant increases of IgG_1_ levels specific for S and RBD (*p* = 0.0002 and *p* = 0.0071, respectively) were found after vaccination. However, no relevant IgG_2_, IgG_3_, or IgG_4_ subclass responses specific for S and RBD could be detected (Figure 1D).

### 2.4. Induction of Virus-Neutralizing Antibodies and Antibodies Inhibiting the RBD-ACE2 Interaction by Gam-COVID-Vac Vaccination

**Figure 2:**
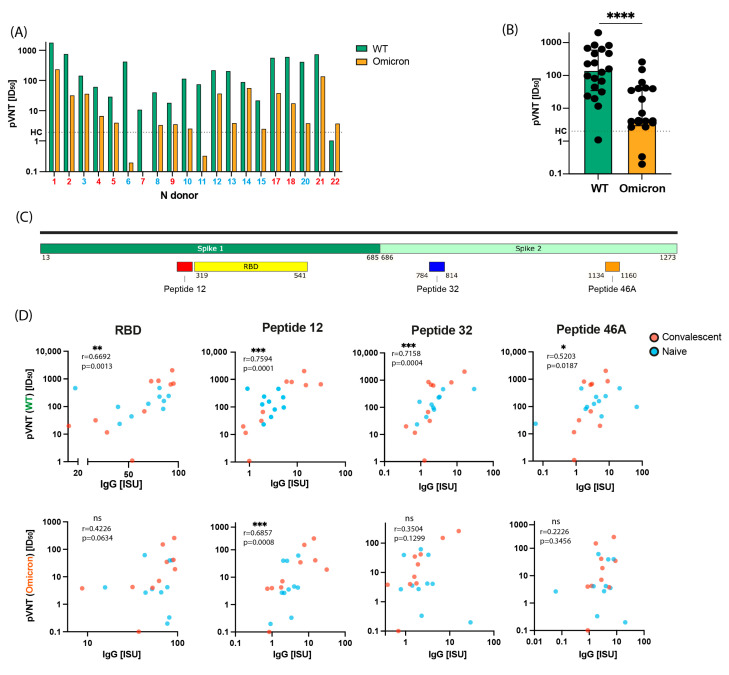
Virus-neutralizing activity in plasma 3 months after Sputnik-V vaccination and association with IgG antibody responses to RBD and S-derived peptides. (**A**) Virus neutralization (ID_50_) (*y*-axis) against Wuhan-hu-1 strain (green) and Omicron (orange) obtained with plasma from naïve (blue) and convalescent (red) subjects (*x*-axes). (**B**) Comparison of virus neutralization (*y*-axis: ID_50_ obtained for plasma from the tested subjects, bars represent medians with interquartile range (IQR) Wuhan-Hu-1 (green) and Omicron (orange). The horizontal line represents results obtained with plasma from a healthy, non-infected control subject (healthy control, HC). Significant differences between strains were determined by Mann–Whitney test: **** *p* < 0.0001. (**C**) Localization of RBD and peptides correlated with virus neutralization in a scheme of S (S1, S2). (**D**) Correlations between virus neutralization titers for Wuhan-Hu-1 (WT) or Omicron (*y*-axes) and IgG levels specific for RBD and spike-derived peptides 12, 32, and 46A (*x*-axes: ISU). Blue and red symbols indicate naïve and convalescent subjects, respectively. Asterisks indicate significant correlations according to nonparametric Spearman test, * *p* < 0.05, ** *p* < 0.01, *** *p* < 0.001 **** *p* < 0.0001, ns = not significant.

For the detection of virus neutralizing antibodies, we used a pVNT test, where virus-like particles were pseudo-typed with S-protein from the original Wuhan-Hu-1 strain and the recently described VOC Omicron. Antibodies with neutralizing activity for Wuhan-Hu-1 strain were found in all but one (i.e., subject 22) of the 22 subjects (Figure 2A, Table 2) whereas neutralizing antibody titers for Omicron were significantly lower (Figure 2B) and found only in 17 out of 20 subjects (Figure 2A, Table 2). No relevant neutralization was found for subjects 6, 7, 10, 11, 15 (Figure 2A, Table 2). Virus neutralization titers for Omicron were 25 times lower than for WT (median 141.9 and 5.7 for Wuhan-Hu-1 and Omicron, respectively) (Figure 2B).

The fact, that in all but one subject (i.e., subject 22) virus neutralizing antibodies increased after vaccination indicates that they were induced by vaccination (Table 2). Subject 21 showed already a high blocking of the RBD-ACE2 interaction in MIA and high virus neutralization at baseline (Table 2). Notably, plasma samples from Gam-COVID-Vac-vaccinated convalescent subjects who had moderate disease (#1, 2, 17, 18, 21) had higher virus neutralization titers than those from convalescent subjects who had mild disease (#22) or no symptoms (#4, 5, 7, 9) (Table 1 and Table 2).

However, a key finding was that inhibition of RBD binding to ACE2 and virus neutralization was not always associated. A more than 25% inhibition of binding of RBD Wuhan-Hu-1 to ACE2 occurred only for 6 subjects who had also shown inhibition in the virus neutralization test due to vaccination (i.e., subjects 1, 2, 3, 17, 18, and 19) (Table 2). Interestingly, virus neutralizing activity (50% infectious dose (ID_50_) > 100) was observed for 6 subjects although they showed no or poor inhibition of RBD binding to ACE2 (Table 2: Subjects 6, 10, 12, 13, 16, 20).

### 2.5. Virus Neutralization in Gam-COVID-Vac-Vaccinated Subjects Is Strongly Correlated with IgG Responses to RBD and to the S-Derived Peptide 12

As indicated before, plasma from several Gam-COVID-Vac-vaccinated subjects (e.g., in subjects 6, 10, 12, 13, 16, 20; Table 2) showed virus neutralizing activity but did not inhibit the RBD-ACE2 interaction. We therefore analyzed IgG antibody reactivity to the peptides spanning the S protein by micro-array-based chip analysis [13] (Figure S4 and Figure 2C) and correlated peptide-specific IgG responses with virus neutralization of the Wuhan-Hu-1 strain and Omicron (Figure 2D). IgG responses to three peptides (i.e., peptides 12, 32, 46A) such as IgG responses to folded RBD correlated significantly with neutralization of the Wuhan-Hu-1 strain (Figure 2C,D).

**Figure 3:**
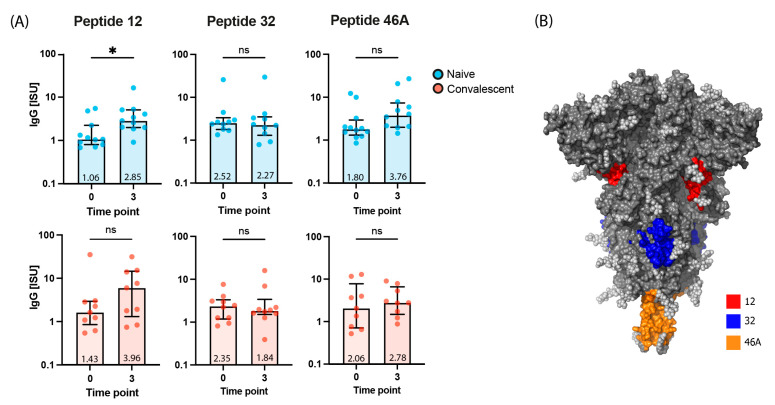
Median IgG antibody levels (y-axes: ISU) to S-derived peptides (P12, P32, P46A) in vaccinated naïve and convalescent subjects at time points 0 and 3 (*x*-axes) (**A**). Significant differences between groups as determined by Wilcoxon test are indicated: * *p* < 0.05, ns = not significant. (**B**) Visualization of peptides 12, 32, and 46A in the surface representation of the SARS-CoV-2 spike protein trimer (side view, pre-fusion conformation) generated with PyMOL. Peptide 12 is highlighted in the S-trimer model in red, peptide 32 in blue, and 46A in orange. Carbohydrates are shown in light grey.

**Figure 4:**
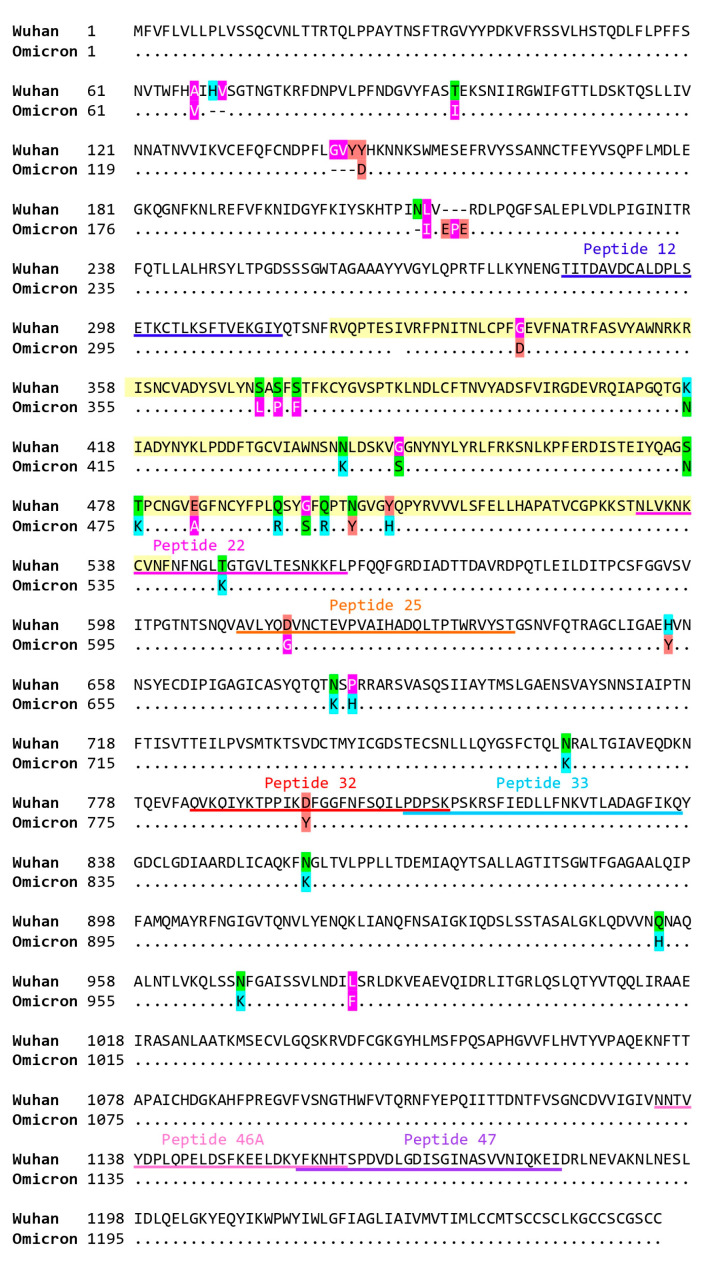
Protein sequence alignment of Wuhan-Hu-1 (top) and Omicron (below) of SARS-CoV-2 S-protein. RBD is colored in light yellow and peptides 12, 22, 25, 32, 33, 46A and 47 are underlined. Wuhan S-protein sequence corresponds to WA1 isolate (GeneBank accession Nr.: BCN86353.1), Omicron S-protein sequence Omicron is clade GRA, lineage B.1.1.529 BA.1 based on the GISAID data base (https://www.gisaid.org/ (accessed on 15 March 2022)). Amino acid substitutions are color-coded according to their hydropathy and charge properties (pink = hydrophobic, green = neutral, light blue = basic hydrophilic, and light red = acidic hydrophilic).

Peptide 12 stood out because antibody responses to unfolded peptide 12 (Figure S1), but not to unfolded peptides 32 and 46A were significantly boosted by Gam-COVID-Vac in naive subjects (Figure 3A) and also significantly correlated with neutralization of Omicron (Figure 2D). No significant boosting was observed for convalescent subjects to peptides 12, 32, or 46A (Figure 3A). The sequence of peptide 12 is highly conserved among SARS-CoV-2 strains (Figure 4) and resides in S1 close to the N-terminus of RBD and hence denotes a region which may be relevant for the transition of the pre- to the post-fusion conformation of the S protein (Figure 3B).

## 3. Discussion

The vector-based vaccine Gam-COVID-Vac also termed Sputnik-V, was one of the first SARS-CoV-2 vaccines which became available for use in clinical practice. However, relatively little information is available regarding the fine-specificities of antibody responses in subjects who have been vaccinated with Gam-COVID-Vac. In this study we performed a detailed analysis of antibody responses towards micro-arrayed folded and unfolded SARS-CoV-2 proteins and peptides spanning the S protein in plasma samples from SARS-CoV-2-naïve and convalescent subjects after a full course of immunizations with Gam-COVID-Vac. Gam-COVID-Vac induced mainly IgG_1_ but no other IgG subclass responses to folded S, S1, S2, and RBD in a comparable manner in naïve and convalescent subjects, indicating that it can be used to boost SARS-CoV-2-specific antibody responses also in convalescent subjects. Like other genetic vaccines, the Gam-COVID-Vac-induced antibody response resided mainly in the IgG_1_ subclass which has a shorter half-life as compared to an IgG_4_ subclass response which is more sustainable. For example, specific IgG_4_ subclass responses are associated with long-term protection during allergen-specific immunotherapy whereas specific IgG_1_ responses build up quickly but fade after a few months [28,29,30]. Accordingly, frequent booster injections of Gam-COVID-Vac and other genetic vaccines will be needed to keep neutralizing antibody responses at a high level. Vaccination with Gam-COVID-Vac induces an antibody response which mimics to a large extent that found after natural infection with SARS-CoV-2 [13]. In addition, antibody responses after natural infection belong mainly to the IgG_1_ subclass and are directed against folded S, S1, S2, and RBD [13]. The novel and interesting finding of our study was that plasma from several Gam-COVID-Vac -vaccinated subjects (e.g., in subjects 6, 10, 12, 13, 16, 20; Table 2) showed virus neutralizing activity but did not inhibit the RBD-ACE2 interaction . We therefore analyzed IgG antibody reactivity to the peptides spanning the complete S protein by micro-array-based chip analysis [13] (Figure 2D and Figure S4) and correlated peptide-specific IgG responses with virus neutralization of the Wuhan-Hu-1 strain and Omicron BA.1 (Figure 2D). IgG responses to three peptides (i.e., peptides 12, 32, 46A) such as IgG responses to RBD correlated significantly with neutralization of the Wuhan-Hu-1 strain (Figure 2C,D). However, peptide 12 was unique because antibody responses only to peptide 12, but not to peptides 32 and 46A were significantly boosted by vaccination (Figure 3A) and also significantly correlated with neutralization of Omicron (Figure 2D). The magnitude of vaccine-induced boosting of IgG responses to peptide 12 was modest but significant in naïve subjects and quite comparable to the boosting of IgG antibodies directed to S and S1. However, IgG antibodies against the isolated unfolded peptide (Figure S1) may represent only a portion of antibodies directed against peptide 12 in the folded protein. Furthermore, not only the levels but also the avidity of the antibodies are important. The sequence of peptides 12 is highly conserved among the Wuhan-Hu-1 strain and BA.1 Omicron VOC (Figure 4) and is derived from a region which may be relevant for the transition of the pre- to the post-fusion conformation of the S protein (Figure 3B). Moreover, the comparison of new mutant variants of Omicron (BA.1, BA.2, BA.4/5, BA.2.12.1, BA.2.75, BQ.1), shows that peptide 12 (284–313 aa.) remains unchanged in terms of amino acid sequence, in contrast to, for example, peptide 32 (https://covariants.org/shared-mutations, (accessed on 28 November 2022)). Notably, monoclonal antibodies with virus-neutralizing ability targeting the N-terminal domain (NTD) region of which peptide 12 is a part thereof have been described [31], and it was reported that also other vaccines can induce an antibody response directed against this region [18,19,20,32], indicating that the region defined by peptide 12 may be important for inducing neutralizing antibodies.

One may therefore assume that antibodies induced by Gam-COVID-Vac vaccination can neutralize SARS-CoV-2 by two mechanisms, one simply by inhibiting the RBD–ACE2 interaction and another by targeting the region containing peptide 12 of the S-protein which may be involved in structural changes (i.e., transition of the pre-fusion into the fusion conformation of S).

Our study has limitations. For example, we used a neutralization assay based on pseudo-typed lentiviruses but these assays are broadly used and reliable [33]. It is also a limitation of our study that peptides on our micro-array were unfolded and thus may have identified only a fraction of neutralizing antibodies which recognized the unfolded peptides whereas an even larger fraction of antibodies may have been bound to conformational epitopes in the identified region. Furthermore, it is a limitation of our study that we analyzed only a small number of subjects. However, our study clearly indicates that antibodies with different epitope specificity may contribute to the protective effect of Gam-COVID-Vac vaccination. In addition, the study shows a response to a potentially conserved peptide that can be used to create broadly neutralizing antibodies.

## 4. Materials and Methods

### 4.1. Study Population and Ethics Statement

Healthy subjects with (n = 10) or without (n = 12) prior SARS-CoV-2 infections were enrolled in the study in December 2020 at the National Research Center Institute of Immunology of The Federal Medical Biological Agency of Russia (Ethics number: #12-1, 29 December 2020). All participants provided written informed consent. Previous SARS-CoV-2 infection was either confirmed by a former positive PCR test and symptoms of COVID-19 or by presence of SARS-CoV-2-specific antibodies directed to nucleocapsid antigen and/or RBD and/or S antigen as described [13]. All subjects were immunized intramuscularly with two doses of Sputnik-V (Gam-COVID-Vac, Biocard, Moscow, Russia) at an interval of three weeks. Blood samples were obtained one day before the first vaccination (T0, Figure 1A) and 80 to 85 days after the first vaccination (T3, Figure 1A).

### 4.2. Detection of SARS-CoV-2-Specific Antibody Responses

Immunoglobulin response of naïve and COVID-19 convalescent subjects was assessed in plasma samples before and after immunization with Sputnik-V (Figure 1A). Specific IgG to a comprehensive panel of SARS-CoV-2 proteins and 46 peptides (25–30 mers) spanning the S protein was measured in 1: 50 diluted plasma samples by microarray-chip technology as previously described [13]. The amino acid sequences, the number of amino acids, and the molecular weight for all synthetic peptides on the micro-arrayed chip are presented in Table 3. For peptides 12 and 32 and RBD expressed in *Escherichia coli* (*E. coli*) (unfolded) and in Human Embryonic Kidney 293 (HEK293) cells (folded) circular dichroism (CD) spectra are provided (Figure S1). Peptides 12 and 32 revealed no specific secondary structure as determined by circular dichroism (CD) as shown in Figure S1.

In detail, glass slides containing microarrays surrounded by an Epoxy frame (Paul Marienfeld GmbH & Co. KG, Lauda-Königshofen, Germany) were activated with an amine-reactive complex organic polymer, MCP-2 (Lucidant Polymers, Sunnyvale, CA, USA) in order to facilitate the binding of proteins and peptides. SARS-CoV-2 antigens/peptides were spotted using a concentration of 0.5–1 mg/mL in (75 mM Na_2_HPO_4_, pH = 8.4) in triplicates using a SciFlexArrayer S12 (Scienion AG, Berlin, Germany) [13]. The expression and purification of proteins and peptide synthesis is described in [13]. IgG antibody reactivity to micro-arrayed antigens was measured by washing first the microarrays for 5 min with phosphate-buffered saline with 0.5% Tween 20 (PBST) and drying them by centrifugation using a Sigma 2–7 centrifuge and MTP-11113 rotor (both Sigma Laborzentrifugen GmbH, Osterode am Harz, Germany). Then 35 µL aliquots of 1:50 diluted plasma samples (sample diluent, Thermofisher, Waltham, MA, USA) were added and incubated for 2 h at 22 °C. After another washing step, 30 µL of secondary antibodies (DyLight 550 (Pierce, Rockford, IL, USA) labeled anti-human IgG (Jackson ImmunoResearch Laboratories, West Grove, PA, USA)) were applied and incubated for 30 min at 22 °C RT. Chips were then washed, dried and subsequently scanned using a confocal laser scanner (Tecan, Männedorf, Switzerland). Image analysis was performed by MAPIX microarray image acquisition and analysis software (Innopsys, Carbonne, France) and conversion of measured fluorescence units to ISAC standardized units (ISU) was performed as described [13]. Specific antibody levels are expressed in ISAC standardized units (ISU). S and RBD-specific IgG_1–4_ antibody responses were measured in plasma samples (1:50 diluted) in duplicates by ELISA as previously described [13] with a variation of <5% for their average values.

### 4.3. Far UV Circular Dichroism (CD) Spectra

For peptides P12 and P32 as well as RBD expressed in *E. coli* and in HEK293 cells CD analysis was performed using a Jasco J-180 spectropolarimeter (Japan Spectroscopic Co., Tokyo, Japan) previously described [34]. CD spectra of peptides and proteins (Figure S1) were measured at a concentration of 0.1 mg/mL in 10 mM NaH_2_PO_4_ (pH 8.0), which was used for spotting of the antigens.

### 4.4. Pseudo-Virus-Based Virus Neutralization Assay (pVNT)

Virus-neutralization activity in plasma samples was determined by pseudo-virus-based virus neutralization assay as previously described [35]. To produce SARS-CoV-2 spike virus-like particles (VLPs), HEK293T cells were co-transfected with 3 plasmids: lentiviral packaging plasmid pCMVΔ8.2R (Addgene, Teddington, UK), pUCHR-GFP, and a pCAGGS-SΔ19 plasmid encoding the wild-type SARS-CoV-2 spike protein (identical to the reference Wuhan-Hu-1 and WA1 isolates) or pCAGGS-SΔ19-Om plasmid encoding the SARS-CoV-2 Omicron spike protein (kind gift from Andrey Gorchakov, Laboratory of Immunogenetics, Institute of Molecular and Cellular Biology, Siberian Branch of the Russian Academy of Sciences, Novosibirsk, Russia). Before use in pVNT, VLPs were titrated by limiting dilution and incubated for 4 days with hACE2-transfected HEK293T cells. A dose of viral particles which gave 50% green fluorescent protein (GFP)-positive cells was selected for use in the test. For pVNT, all plasma samples were heat-inactivated for 30 min at 56 °C prior to use. On the 4th day, post-infection cells were re-suspended, and the percentage of GFP-positive cells was measured by flow cytometry. ID_50_ values were calculated using a sigmoidal curve (GraphPad Prism 9.2.0 software, Sigmoidal, 4PL), reconstructed by the percentage of neutralization at the different indicated plasma concentrations.

### 4.5. Molecular Interaction Assay (MIA)

To detect the capacity of plasma samples of COVID-19 convalescent or naïve subjects (Table 1), prior and after Sputnik-V immunization, to inhibit the binding of 50 ng/mL of RBD-hu-1 to ACE2 receptor a molecular interaction assay (MIA) was performed as described [13,36,37,38,39]. This assay can measure the inhibition of labelled RBD to ELISA plate-bound ACE2 by antibodies or other compound inhibition of the RBD-ACE2 interaction [38]. In brief, recombinant ACE-2 (GenScript, Piscataway, NJ, USA) was coated (2 µg/mL) overnight onto NUNC Maxisorb 96 well plates (Thermofisher). Thereafter, three washing cycles were conducted with washing buffer and then plates were blocked for 3 h at RT with blocking buffer. Serum samples were diluted 1:2 in PBS, 0.05% Tween 20, 1% BSA, and pre-incubated for 2 h with 50 ng His-tagged recombinant RBD (GenScript). Then the pre-incubated serum samples were added to the plates containing ACE-2 for 3 h and the plates were washed and incubated overnight with a 1:1000 diluted mouse monoclonal anti-His tag antibody (Dianova, Hamburg, Germany). After 3 washing steps, 1:1000 diluted HRP-linked anti-mouse IgG1 antibody (GE Healthcare, Chicago, IL, USA) was added for 2 h and detection was performed with ABTS. Mean optical density (O.D.) values corresponding to bound RBD were measured at 405 nm and 492 nm (reference) in a TECAN Infinite F5 ELISA reader with the integrated software i-control 2.0 (Tecan Group Ltd., Männedorf, Switzerland). The buffer control (overlay without RBD) was subtracted from each result. Determinations were performed in duplicates and results are shown as mean values with a variation of <5%.

### 4.6. Visualization of Peptides in the Spike Protein Structure

Surface representation of SARS-CoV-2 spike protein was generated in PyMOL (PyMOL Molecular Graphics System, Version 2.5.0a0, Schrödinger, LLC, New York, NY, USA) based on the PDB entry 6XR8.

### 4.7. Statistical Analysis

Statistical analysis was performed using GraphPad Prism (version 9.2.0 GraphPad Software, La Jolla California). The Wilcoxon test (Figure 1C,D and Figure 3A), Mann–Whitney test (Figure 2B and Figure S3), and Friedman test (Figure S2) were used for comparison between two or multiple groups. *p* < 0.05 was considered statistically significant. The correlation between two groups was determined by the Spearman rank test (Figure 2D). A normalized non-linear regression was performed using GraphPad Prism software (Sigmoidal 4PL). Data are presented as median ± IQR. Asterisks indicate significant difference between groups, * *p* < 0.05, ** *p* < 0.01, *** *p* < 0.001, **** *p* < 0.0001, ns—not significant.

## 5. Conclusions

In summary, our study is the first to study in depth the epitope and IgG subclass specificity of antibodies induced by Gam-COVID-Vac and to reveal different mechanisms of Gam-COVID-Vac-induced virus neutralization by antibodies. In particular we obtained evidence that antibodies directed to a peptide (i.e., peptide 12) located close to but outside of RBD in the N-terminal part of S1 are associated with virus neutralization. The potential virus-neutralizing activity of peptide 12-specific antibodies may be due to their ability to interfere with the transition of the pre- to the post-fusion conformation of the spike protein. We suggest consideration of inclusion of the region defined by peptide 12 in an immunogenic form in vaccines to induce antibodies against this epitope area and to test antibodies induced by such vaccines for virus neutralization. In the case that virus neutralization of vaccines can be enhanced, inclusion of an immunogenic peptide 12 epitope may improve the efficacy of COVID-19 vaccines. In addition, one may consider developing antibodies against the peptide 12-defined epitope for passive immunization. Due to the fact that the peptide 12-defined epitope is conserved in currently known SARS-CoV-2 variants, vaccines and antibodies directed to peptide 12 may cross-protect against SARS-CoV-2 variants. We thus provide novel knowledge which may be helpful for the development of novel active and passive vaccination strategies for COVID-19.

## Figures and Tables

**Table 1:** Demographic, clinical, and serological characteristics of subjects.

	Subjects	Sex	Age	Polymerase Chain Reaction (PCR)-Confirmed COVID-19 Symptoms (before Vaccination)	PCR-Confirmed COVID-19 Symptoms (Days after Vaccination)	Nucleocapsid-Specific IgG at Baseline (ISAC Standardized Units (ISU))	RBD-Specific IgG at Baseline (ISU)
Convalescent	1	M	61	−70 to −61	no	35.6	20.1
	2	M	69	−53 to −46	no	93.6	49.6
	4	F	60	no	no	1.9	16.8
	5	M	60	no	no	25.6	6.2
	7	F	66	no	no	9.2	3.4
	9	M	66	no	no	12.6	2.9
	17	M	42	−86 to −78	no	19.8	43.0
	18	M	61	−120 to −110	no	23.8	22.5
	21	F	53	−88 to −71	no	8.9	68.0
	22	F	53	no	110 to 128	0.8	45.5
Naive	3	F	62	no	no	2.0	0.7
	6	M	70	no	no	3.8	0.8
	8	F	54	no	no	5.3	5.9
	10	F	43	no	no	7.7	0.5
	11	F	61	no	no	1.2	2.1
	12	F	52	no	no	3.5	1.0
	13	F	25	no	no	1.8	0.9
	14	F	46	no	no	3.4	0.7
	15	F	43	no	no	1.0	1.5
	16	F	51	no	no	0.8	0.5
	19	F	66	no	no	0.4	0.5
	20	F	62	no	no	0.8	0.9

**Table 2:** Comparison of IgG levels specific for S, S1, S2, and RBD measured by micro-array with inhibitions of RBD binding to ACE2 and virus neutralization titers (Wuhan-Hu-1, Omicron) determined for plasma from the investigated subjects. Heat mapping was performed for each parameter separately using Excel. The highest level was considered dark red and the lowest white.

Subject ID	Time Point	S (Folded) [ISU]	S1 (Folded) [ISU]	S2 (Folded) [ISU]	RBD (Folded) [ISU]	N-Protein [ISU]	Inhibition of RBD-ACE2 [%]	pVNT (Wuhan) [ID_50_]	pVNT (Omicron) [ID_50_]
1	0	1.434	18.549	0.799	20.124	35.639	−6.67	37.78	3.6
	3	13.031	36.751	10.887	91.877	12.119	99.36	2014.01	258.8
2	0	2.239	5.195	0.983	49.559	93.621	2.05	80.46	1.49
	3	0.384	21.944	4.731	75.926	42.004	94.92	845.2	34.81
3	0	0.153	0.977	0.391	0.693	1.979	3.38	0.91	0.52
	3	16.509	19.899	1.959	81.847	1.263	37.07	158.2	39.41
4	0	0.126	4.534	0.424	16.791	1.891	2.01	0.01	0.34
	3	10.17	13.711	1.685	62.309	0.866	5.07	66.89	7.12
5	0	0.214	0.857	1.501	6.184	25.591	1.21	0.01	1.22
	3	4.364	5.291	3.503	31.586	26.784	5.26	31.43	4.25
6	0	0.295	6.772	0.469	0.784	3.793	−1.22	2.5	0.23
	3	3.045	13.996	2.777	76.984	6.099	−26.45	468.64	0.2
7	0	0.22	4.933	0.546	3.362	9.165	1.83	0.72	0.12
	3	2.006	7.43	1.611	37.039	2.497	12.04	11.57	0.1
8	0	0.161	2.737	0.382	5.921	5.293	7.24	0.07	2.45
	3	6.624	12.456	1.465	52.134	0.889	−6.91	43.82	3.575
9	0	0.12	3.664	0.237	2.907	12.566	−1.25	0.01	1.12
	3	0.59	3.497	0.219	8.714	2.664	23.59	19.68	3.797
10	0	0.07	3.209	0.497	0.486	7.663	−12.19	0.08	0.17
	3	8.034	10.476	1.369	65.884	1.248	8.6	125.56	2.713
11	0	0.308	2.071	0.371	2.141	1.206	13.39	2.81	0.54
	3	13.721	17.25	3.061	81.083	1.476	−10.69	81.45	0.3323
12	0	0.287	1.171	2.658	1.017	3.53	−2.96	4.91	1.03
	3	16.199	17.508	3.85	87.431	3.791	−4.49	241.16	39.98
13	0	0.414	3.224	0.38	0.929	1.836	0.01	0.91	0.06
	3	4.931	11.493	1.107	76.989	0.716	−14.85	226.12	4.169
14	0	0.103	6.739	1.6	0.651	3.365	2.36	2.43	0.15
	3	2.533	7.719	1.5	43.182	1.09	−1.21	96.93	61.77
15	0	0.136	2.887	1.739	1.5	1.004	−17.01	1.39	2.34
	3	3.139	6.521	0.626	44.147	0.431	−2.66	23.53	2.67
16	0	0.179	1.581	0.541	0.535	0.786	−7.95	1.27	2.77
	3	6.161	11.499	3.866	70.174	8.907	−8.25	506.21	24.31
17	0	4.492	6.83	2.311	43.009	19.826	−13.41	78.81	2.58
	3	26.607	29.214	4.489	90.084	6.7	35.58	631.3	41.34
18	0	2.111	3.474	0.741	22.527	23.754	8.37	65.39	3.1
	3	10.915	38.111	5.796	93.621	10.836	23.64	673.21	18.92
19	0	0.311	1.259	0.3	0.466	0.374	7.24	13.14	6.87
	3	12.446	35.571	15.249	78.687	11.299	99.6	780.4	188.4
20	0	0.32	11.647	1.021	0.92	0.819	4.95	3.93	2.6
	3	1.763	6.95	0.584	15.657	0.552	−3.68	460.98	4.1
21	0	0.35	46.049	25.096	68.013	8.907	98.49	1224.01	109.6
	3	10.709	22.227	5.884	68.567	0.104	98.97	823.67	150.6
22	0	0.385	1.607	0.281	45.468	0.828	4.46	0.08	0.5
	3	1.5	4.581	0.3	52.691	0.834	−3.33	1.09	4

**Table 3:** Synthetic SARS-CoV-2 spike protein-derived peptides ^§^.

Peptide	Amino Acid Sequence	Molecular Weight [Da]	No. of Amino Acids
1	PLVSSQCVNLTTRTQLPPAYTNSFTRGVYY	3377.8	30
2	RGVYYPDKVFRSSVLHSTQDLFLPFFSNVT	3520.9	30
3	FSNVTWFHAIHVSGTNGTKRFDNPVLPFND	3418.7	30
4	LPFNDGVYFASTEKSNIIRGWIFGTTLDS	3249.6	29
5	TLDSKTQSLLIVNNATNVVIKVCEFQFCND	3357.8	30
6	QFCNDPFLGVYYHKNNKSWMESEFRVYSSA	3648.0	30
7	VYSSANNCTFEYVSQPFLMDLEGKQGNFKN	3431.8	30
8	GNFKNLREFVFKNIDGYFKIYSKHTPINLV	3603.1	30
9	PINLVRDLPQGFSALEPLVDLPIGINITR	3171.7	29
10	NITRFQTLLALHRSYLTPGDSSSGWTAGAA	3192.5	30
11	TAGAAAYYVGYLQPRTFLLKYNENGTITDA	3282.6	30
12	TITDAVDCALDPLSETKCTLKSFTVEKGIY	3262.7	30
13	EKGIYQTSNFRVQPTESIVRFPNITNLC	3255.7	28
14	FNATRFASVYAWNRKRISNCVADYS	2940.2	25
15	VADYSVLYNSASFSTFKCYGVSPTK	2735.0	25
16	VSPTKLNDLCFTNVYADSFVIRGDEVRQIA	3371.8	30
17	VRQIAPGQTGKIADYNYKLPDDFTGCVIAW	3340.8	30
18	CVIAWNSNNLDSKVGGNYNYLYRLFRKSNL	3522.9	30
19	KPFERDISTEIYQAGSTPCNGVEGF	2746.0	25
20	GVEGFNCYFPLQSYGFQPTNGVGYQPYRVV	3387.7	30
21	PYRVVVLSFELLHAPATVCGPKKSTNLVKN	3281.9	30
22	NLVKNKCVNFNFNGLTGTGVLTESNKKFL	3200.7	29
23	PFQQFGRDIADTTDAVRDPQTLEILDIT	3176.4	28
24	ILDITPCSFGGVSVITPGTNTSNQVAVLY	2967.3	29
25	AVLYQDVNCTEVPVAIHADQLTPTWRVYST	3390.8	30
26	RVYSTGSNVFQTRAGCLIGAEHVNNSYECD	3291.5	30
27	SYECDIPIGAGICASYQTQTNSP*RRAR*SVA	3215.5	30
28	TMSLGAENSVAYSNNSIANNSIAIPTNFTI	3115.4	30
30	TSVDCTMYICGDSTECSNLLLQYGSFCTQL	3296.7	30
31	FCTQLNRALTGIAVEQDKNTQEVFAQVKQI	3393.8	30
32	QVKQIYKTPPIKDFGGFNFSQILPDPSK	3193.6	28
33	PDPSKPSKRSFIEDLLFNKVTLADAGFIKQ	3362.8	30
34	GFIKQYGDCLGDIAARDLICAQKFNGLTVL	3243.7	30
35	TDEMIAQYTSALLAGTITSGW	2229.4	21
36	ITSGWTFGAGAALQIPFAMQMAYRFNGIGV	3176.7	30
37	NGIGVTQNVLYENQKLIANQFNSAIGKIQD	3290.6	30
38	GKIQDSLSSTASALGKLQDVVNQNAQALNT	3072.3	30
39	LNTLVKQLSSNFGAISSVLNDILSRLDK	3046.5	28
40	LDKVEAEVQIDRLITGRLQSLQTYVTQQ	3245.6	28
41	YVTQQLIRAAEIRASANLAATKMSECVL	3051.5	28
42	CVLGQSKRVDFCGKGYHLMSFPQSAPH	2993.4	27
43	PHGVVFLHVTYVPAQEKNFTTAPAICHDGK	3277.7	30
44	CHDGKAHFPREGVFVSNGTHWFVTQRNFYE	3566.9	30
45	RNFYEPQIITTDNTFVSGNC	2319.5	20
46	NNTVYDPLQPELDSFKEELDKYFKNHT	3285.5	27
47	FKNHTSPDVDLGDISGINASVVNIQKEI	3011.3	28

^§^ adapted from reference [13] (Gattinger P, Niespodziana K, Stiasny K, et al. Neutralization of SARS-CoV-2 requires antibodies against conformational receptor-binding domain epitopes. Allergy. 2022;77(1):230-242. doi:10.1111/all.15066). Peptide 29 could not be synthesized and purified.

## Data Availability

The data that support the findings of this study are available from the corresponding author upon reasonable request.

## Supplementary Materials

The following supporting information can be downloaded at: https://www.mdpi.com/article/10.3390/ijms24065104/s1. Reference [39] is cited in the Supplementary Materials.
