# Supplementary material for: Dissection of Antibody Responses of Gam-COVID-Vac-Vaccinated Subjects Suggests Involvement of Epitopes Outside RBD in SARS-CoV-2 Neutralization
Source: Int J Mol Sci. 2023 Mar 7;24(6):5104. doi: 10.3390/ijms24065104 (PMC10049224; doi:10.3390/ijms24065104)

Figure S1. Circular dichroism analysis of (A) RBD expressed in E. coli or HEK293 cells[§] and (B) synthetic peptides 12 and 32. Molecular ellipticities (y-axes) at given wave lengths (x-axes) are shown.
[§]adapted from [39] (Gattinger et al. 2022, doi: 10.1111/all.15305).

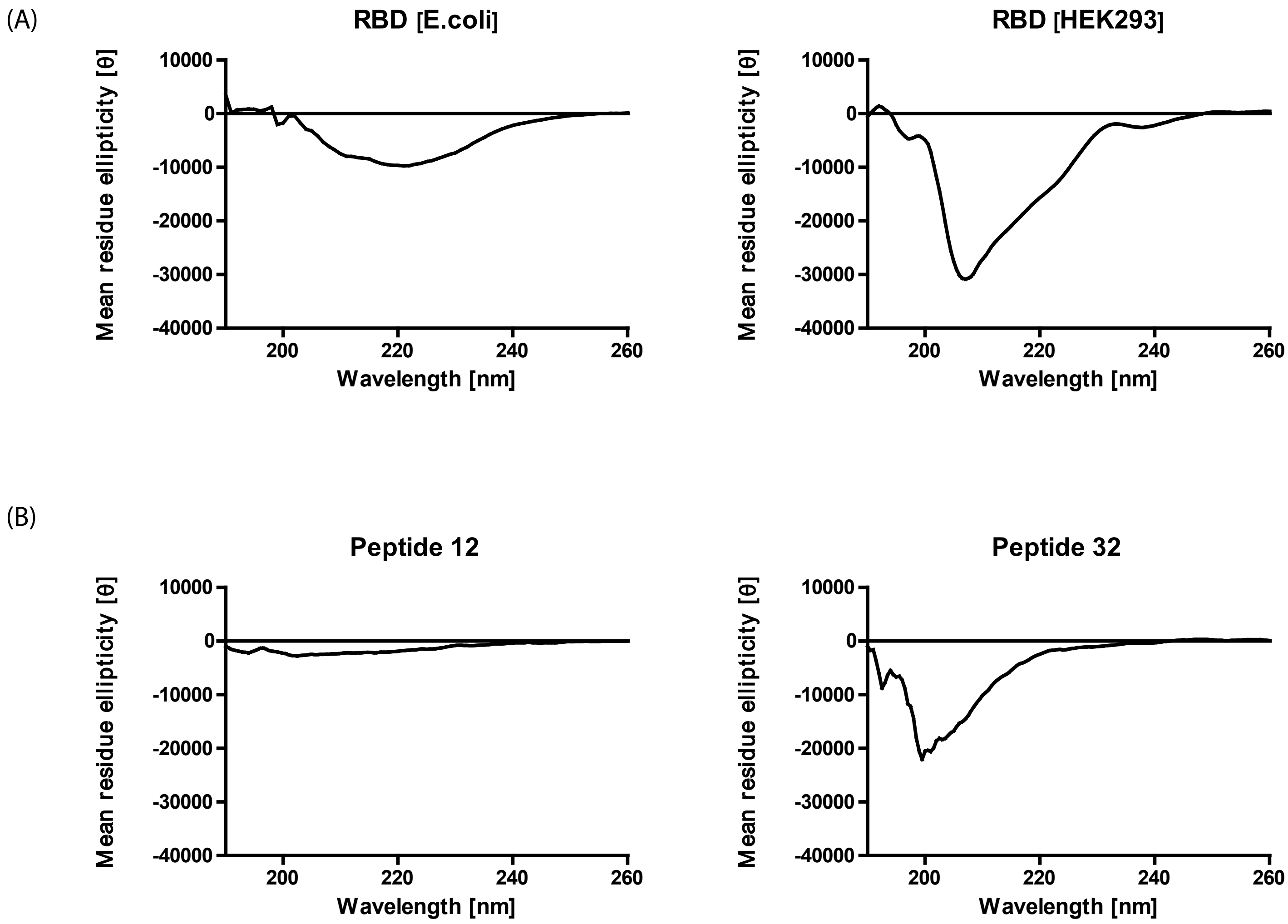

Figure S2. IgG antibody levels (y-axes: ISU) specific for RBD, S, S1 and S2 in naïve (colored in blue) and convalescent (colored in red) subjects at time point 0 and 3. The Friedman test was used for comparison between groups. Asterisks indicate significant difference between groups, *P < 0.05, ***P < 0.001, ns - not significant.

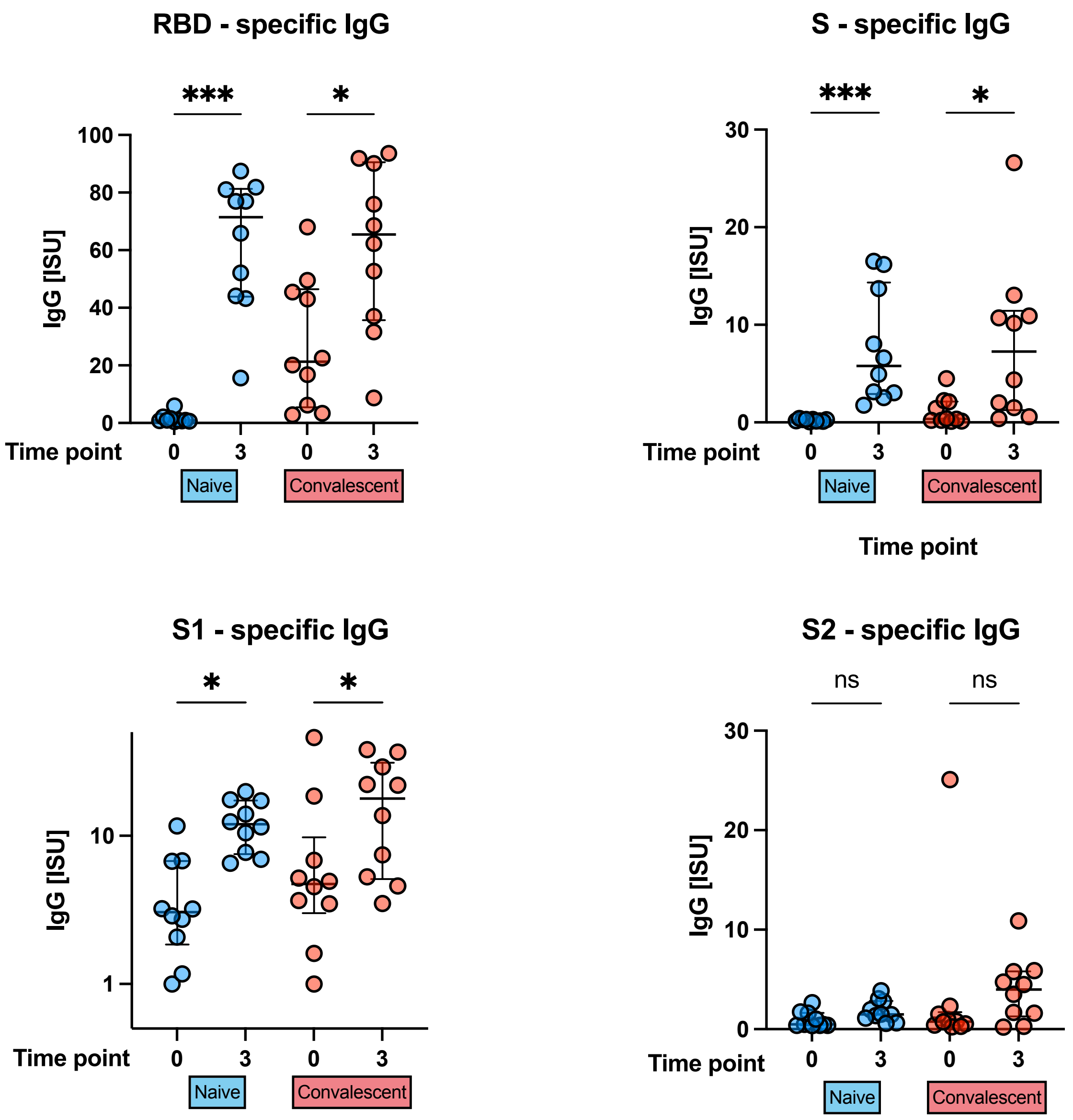

Figure S3. Comparison of IgG antibody levels (y-axes: ISU) specific for RBD, S-protein, S1 and S2 (x-axes) between naïve (colored in blue) and convalescent (colored in red) subjects at time point 3. The Mann-Whitney test was used for comparison between two groups. Asterisks indicate significant difference between groups, ns - not significant.

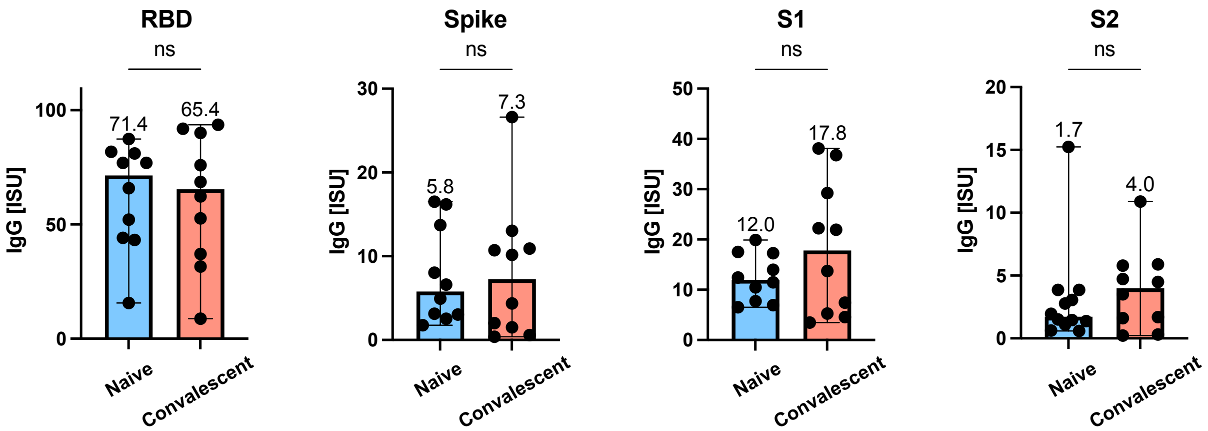

Figure S4. Changes of IgG antibody levels (y-axes: delta mean fluorescent intensities MFI) to S-derived peptides (x-axes) in naïve (lower part) and convalescent subjects (upper part) between time points 0 and 3.

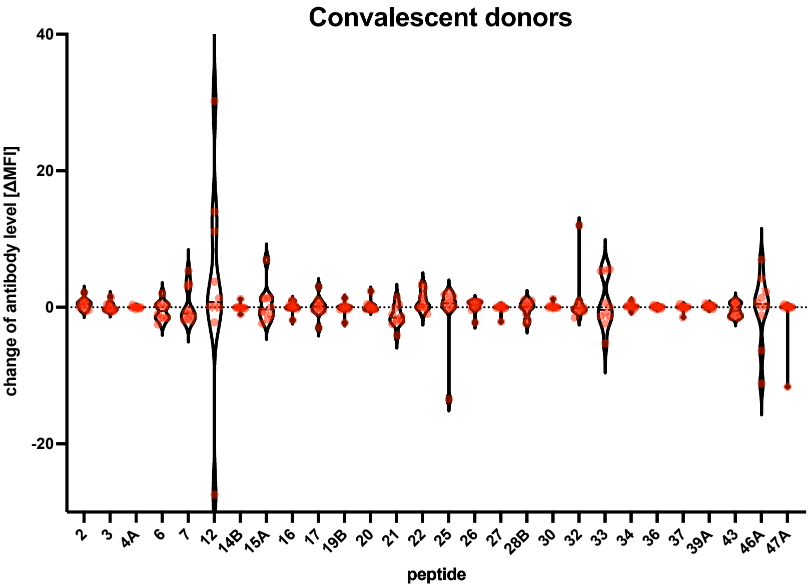

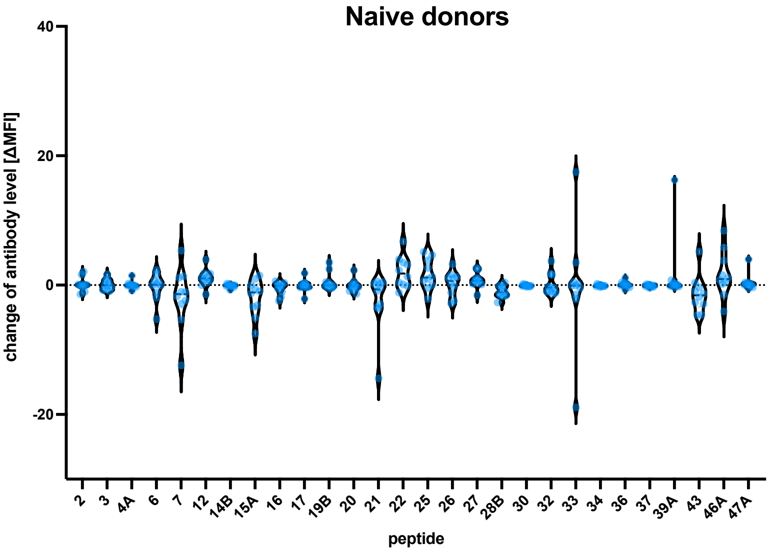

Supplement: Supplementary file 1 [file ijms-24-05104-s001.zip › ijms-2225240-supplementary.pdf]
